# Selected Properties of Formaldehyde-Free Polymer-Straw Boards Made from Different Types of Thermoplastics and Different Kinds of Straw

**DOI:** 10.3390/ma14051216

**Published:** 2021-03-04

**Authors:** Radosław Mirski, Aleksandra Banaszak, Pavlo Bekhta

**Affiliations:** 1 Department of Wood-Based Materials, Poznań University of Life Sciences, 60-627 Poznań, Poland; banaszak@up.poznan.pl; 2 Department of Wood-Based Composites, Cellulose, and Paper, Ukrainian National Forestry University, 79057 Lviv, Ukraine; bekhta@nltu.edu.ua

**Keywords:** polymer-straw boards, thermoplastic polymers, straw, hydrophobicity, mechanical properties

## Abstract

This research investigated the effects of different thermoplastics types and different kinds of straw on selected properties of polymer-straw boards. Polyethylene, polyethylene, and polystyrene of virgin and of recycled origin were used for bonding the boards. Three kinds of straw were used: rape (*Brassica napus* L. var. napus), triticale (*Triticosecale Witt* b m.), and rye (*Secale* L.). Five-layer polymer-straw boards were produced. The obtained boards differed in both the materials they were made of and the moisture content (7, 25, and 2% for the core, the middle, and the face layers, respectively), and 30% of straw particles were substituted with thermoplastics added to the face layers. It was found that properties of polymer-straw boards strongly depend on both the kind of straw and the type of polymer used. The best mechanical properties were obtained for rye straw and polystyrene or recycled polymers, whereas the best hydrophobic properties were observed for rape straw combined with recycled polyethylene or polypropylene. Although recycled polymers improved the hydrophobic properties of the boards, they impaired their mechanical properties in comparison with the reference ones. However, in terms of bending strength, they still met the requirements for heavy duty load-bearing boards for use in humid conditions (20 MPa for P7 boards according to EN 312).

## 1. Introduction

Wood-based materials, especially particleboards, have been greatly popular for many years. The quality of technological lines, excluding those requiring chips of specific type, e.g., OSB (oriented strand board) [1,2,3,4,5,6], enables processing of practically any type of wood into particleboards. Wood generated as waste from sawmill production processes [7,8,9], from forest fires [10], post-consumer or recycled wood, or fine and undersize chips [11,12,13,14,15,16,17,18] are easily used in these processes. This ease of the use of wood material even of low quality does not hinder a search for alternate materials for production of furniture or construction boards. Therefore, works on the possible use of straw from various cereal species for production of particleboards [19,20,21,22,23,24,25,26,27,28,29,30] or even boards as demanding as OSB [31,32,33] are continued. Particles from these straws can partly substitute wood chips or be used as a homogeneous component of manufactured boards. Wheat, rye, rice, or even maize straws are used most commonly. These materials represent an attractive substitute for wood due to a range of useful properties of products, of which they are a main component. For example, they provide better hygroscopicity, better thermal [34] and acoustic [35] insulation, and lower [36] specific weight, and they are much cheaper than wood. Attempts to use straw in particleboards were already made in the 1960s; however the problem of worse gluing of straw particles, when compared with wood, was hard to overcome. Problems with gluing, as well other issues related to urea-formaldehyde (UF) or phenol-formaldehyde (PF) resins, can be solved by using pMDI (polymeric diphenylmethane diisocyanate) adhesive [32,37,38,39]. Straw particles glued with pMDI adhesive and formed into medium- or high-density boards can be successfully used in furniture or construction sectors. In this second industry, these boards can be used for structural applications [30,33,39,40]. However, the company VestaEco uses straw particles glued with pMDI in its technology for production of insulation materials [41].

Particles obtained from straw were also successfully used as a filler for manufacturing of wood plastic composites (WPC) [42,43]. WPCs are commonly manufactured from meal, sawdust, or fine chips by various ways, such as compression molding, extrusion, or injection [44]. These methods ensure better mixing of components, but require much finer fractions of lignocellulosic fillers. The use of methods commonly used for production of wood-based materials offers some solutions to this problem [45,46,47,48,49,50,51,52,53]. In this case, layers of lignocellulosic and thermoplastic materials can be formed alternately or thermoplastic material with particles of a size similar to plant particles can be used [53,54,55,56]. Typical thermoplastic materials for WPC production include polypropylene, polyethylene, polystyrene, or polyvinyl chloride. 

Their advantage is a possibility of their reuse for production of the same or other products. In both cases, products of standard quality are obtained. These materials should be recovered when they become a municipal waste. In the European Union, basic rules and definitions concerning waste management are specified in the Waste Framework Directive [57]. Recovered plastics usually undergo mechanical recycling. However, thermoplastic waste can undergo either material or energy recycling. The energy recycling through incineration can always be the last stage in the cycle of the product life. About 6.3 billion tons of waste plastics are fabricated each year all over the world, while only a slight part of them (~9%) are recycled. Consequently the majority of them (~79%) are accumulated in the environment. [58]. Although it may appear strange, the demand for recycled plastics in Europe represents ca. 6% of the total demand for plastic materials. Therefore, it seems that, with overproduction of straw (it is estimated that, in the UK alone, as much as 4 million tonnes of straw are wasted every year), a direction enabling combining these two materials into a product of advantageous physical and mechanical properties is a correct one for the coming years.

Thus, the presented study aimed to combine wastes of straw and thermoplastics into polymer-straw board and to determine the effect of a kind of straw and a type of thermoplastics on both mechanical and physical properties of manufactured polymer-straw boards.

## 2. Materials and Methods

### 2.1. Materials

Rye (Ys), triticale (Ts), and rape (Rs) straws were used in the study. The straw was obtained in an unshredded form and then shredded in a laboratory mill. An attempt was made to obtain chopped straw of a length similar to pine chips (Pw) used in the middle layer in particleboard production. Particles obtained by shredding were additionally sieved on a screen with 0.5 × 0.5 mm^2^ mesh, to remove very fine and dust fractions. Samples for size and bulk density determinations were collected from prepared batches of the material. Linear dimensions of particles obtained this way are shown in Table 1. A detailed description is provided in publication [29]. Data included in Table 1 shows that obtained particles differ mainly in their thickness. This parameter is difficult to influence during the shredding process. Thickness of grass stalks is much lower than that of rape straw, and this translates into a lower thickness of obtained particles. The linear dimensions influence bulk density of obtained fractions. In this case, fractions for board manufacturing produced by shredding rye and triticale straws had similar bulk density of 60 kg/m^3^, while bulk density of fractions obtained from rape straw was 80 kg/m^3^. Bulk density of pine chips was over twice as high as that of cereal chips.

The following thermoplastic materials, differing in types, forms, and softening point, were used in the study:

- PP—polypropylene,
- HDPE—high density polyethylene,
- LDPE—low density polyethylene,
- PS—polystyrene,
- LDPE rec. yellow—low density polyethylene from recycled scraps of yellow plastic,
- LDPE rec. pink—low density polyethylene from recycled scraps of pink plastic,
- PP recycled—polypropylene from reusable packaging.

In these studies, both native/virgin and recycled polymers were used. Their basic characteristics and appearance are presented in Table 2.

The polymers used in the study are commonly available, and their recycling is not problematic, as they can be reprocessed many times. However, new, alternate methods for their management are continuously sought. When they are combined with wood, the obtained material has better hydrophobic properties than wood. However, the use of adhesion promoters facilitates achieving better biding of wood and polymer. Isocyanate compounds can be used both as agents binding wood or straw particles with each other, and as adhesion promoters for systems composed of lignocellulosic material and thermoplastic polymer. Therefore, we decided to use a polymeric diphenylmethane diisocyanate adhesive (pMDI, Ongronat® 2100, BorsodChem Group, Kazincbarcika, Hungary) in our study. Ongronat® 2100 is a brown (20 °C, 1013 hPa) non-flammable liquid of a density 1.23 g/cm^3^, viscosity of 210 mPa·s, contractual dry matter content of 100%, NCO content of 30.6%, and chlorine hydrolytic 127 mg/kg.

### 2.2. Manufacturing of Polymer-Straw Samples

**Figure 1:**
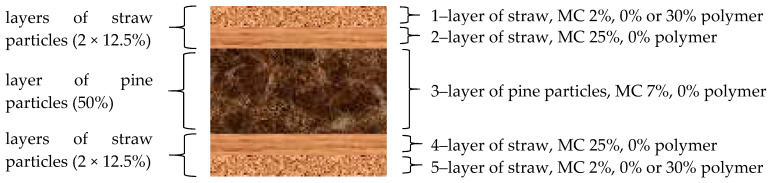
Structure of manufactured boards.

In the laboratory conditions, five-layer boards were produced, of the following shares of individual layers: 12.5%:12.5%:50%:12.5%:12.5%. The individual layers varied in terms of the type of material and moisture content (Figure 1):

- face layers (1, 5) were made of straw of 2% moisture content, with 30% polymer addition,
- intermediate layers (2, 4) were made of straw of 25% moisture content,
- the core layer (3) was made of pine chips of 7% moisture content.

Our previous studies [59] indicate that it is advantageous to use lignocellulosic material of low moisture content, i.e., below 4%, in manufacturing polymer containing products, and this observation is supported by other authors [60,61,62]. For this reason, the lignocellulosic material intended for face layers was dried to a level below 2%. To accelerate the process of heat transfer into the board, intermediate layers were manufactured of material with 25% moisture content. It is consistent with a general practice of particleboard production [63,64,65]. Therefore, the boards produced without thermoplastics were evaluated prior to actual experiments. The following materials were prepared:

- single-layer boards made only of pine chips with 7% moisture content;
- three-layer boards, with a core layer of pine chips and face layers of straw particles of moisture content of ca. 7%;
- five-layer boards of the same structure as the boards with polymers.

Table 3 presents systems of the manufactured boards with symbols assigned.

Thirty percent of the straw were substituted with the thermoplastics which were added to layers 1 and 5. Regardless of the layer, the particles were glued with pMDI adhesive in the amount of 4% of adhesive dry weight per dry weight of the lignocellulosic material. The manually formed mat was prepared to produce the boards with the thickness of 15 mm and the target density of 600 kg/m^3^. The unit pressure was 2.5 MPa, the temperature of heating plate was 200 °C and pressing time was 20 s per mm of the board thickness. Pressing process of the mat was conducted between the metal plates. 

After hot pressing the molds with the metal plates were subjected to the cold pressing, where they were left under 1000 N of load until their temperature was decreased to the range of 80–100°C. The dropping temperature was registered with K type thermocouples attached to both the upper and the lower surfaces of the plates. Three molds with a dimensions of 700 × 450 mm^2^ were produced for each variant. 

### 2.3. Board Testing

After the boards conditioning for seven days at 55 ± 5% RH and 21 ± 1 °C the produced panels were tested in terms of following parameters according to the relevant standards:

- bending strength (MOR) and modulus of elasticity (MOE) according to EN 310 [66];
- internal bond (IB) according to EN 319 [67];
- internal bond after the boiling test (V-100) according to EN-1087-1 [68];
- thickness swelling (TS) after 24 h according to EN 317 [69] and water absorption (WA).

The water resistance and mechanical properties investigations involved from 10 to 16 samples in each variant. The other analysis was conducted in three to five replications.

### 2.4. Statistical Analysis

The results were analyzed using STATISTICA 13.0 package (StatSoft Inc., Tulsa, OK, USA). The performed analysis was based on ANOVA (analysis of variance) and homogeneous groups were distinguished with the use of Tukey’s test (HSD). Homogeneous groups are marked with lowercase letters. The results were analyzed on the significance level of p = 0.05. 

## 3. Results and Discussion

### 3.1. Bending Strength and Modulus of Elasticity in Bending of Board Samples

**Figure 2:**
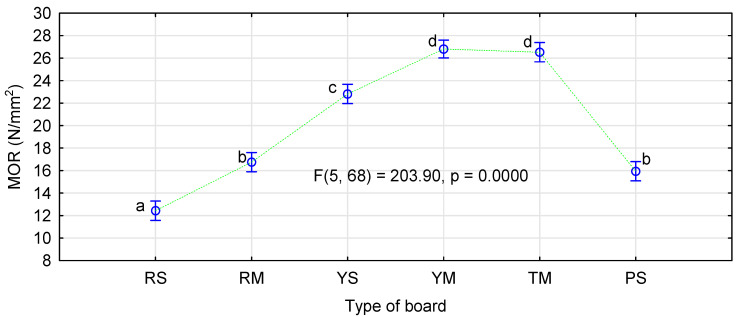
The influence of the board production method and material type on static bending strength: Pl–values for boards containing polymers.

**Figure 3:**
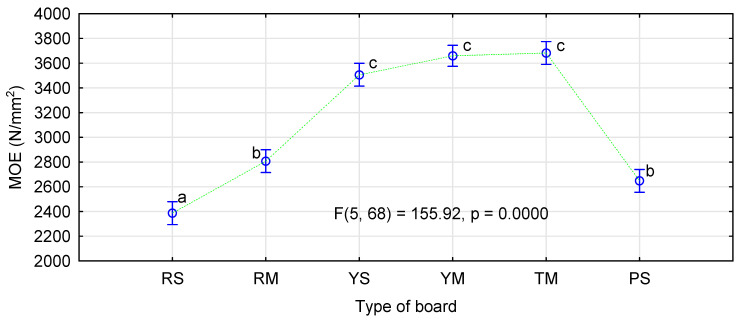
The influence of the board production method and material type on modulus of elasticity.

Figure 2 and Figure 3 show the influence of the board production method on their mechanical properties. The results shown indicate that when a moisture level typical for pMDI-glued boards is used, the lowest MOR and MOE values are observed for the boards made of rape particles. Our previous studies [23,29] indicate that the boards made of rape particles have static bending strength and modulus of elasticity similar to or slightly lower than the boards made of pine chips. The boards made of straw from popular cereals manufactured in the similar conditions are characterized by significantly higher strength and modulus of elasticity than the pine boards [29]. The applied modification, in form of the increased moisture content in the face layers, resulted in a significant increase in bending strength and modulus of elasticity of the rape boards and in bending strength only in the boards made of rye particles.

**Figure 4:**
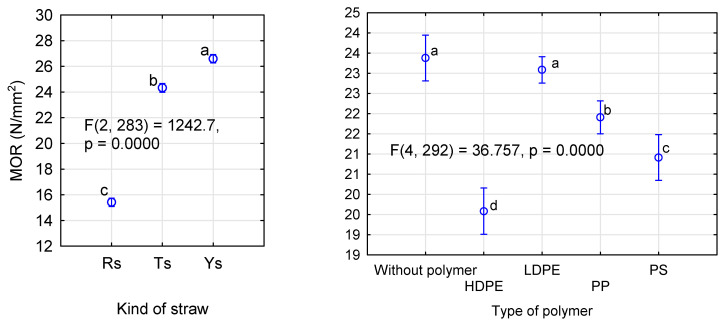
ANOVA for the influence of a straw kind (**left**) and a polymer type (**right**) on static bending strength of polymer-straw boards.

It can be assumed that the use of very dry particles in the face layers does not negatively affect the quality of the manufactured boards. The increase in moisture content in the intermediate layer possibly compensates for the negative effect of excessively dry layers in direct contact with heating panels. The influence of the kind of straw and the thermoplastic type was analyzed by ANOVA. We decided to present more extensive results for this analysis only for the influence of the applied modifications on MOR. Figure 4 shows the influence of the kind of straw in the face layers and the type of polymer used. Similarly as in control samples, the manufactured rape boards differ significantly from the boards made from triticale or rye straw.

Differences in strength of the triticale and rye boards are statistically significant, contrary to results for the systems without polymers (Figure 2). In our previous studies, we also did not find statistically significant differences in strength of the boards made of these straws [29]. In general, all boards containing thermoplastics in their face layers demonstrate lower strength than the control boards. However, in the case of the boards with LDPE in their face layers, this decrease in strength is not statistically significant.

**Figure 5:**
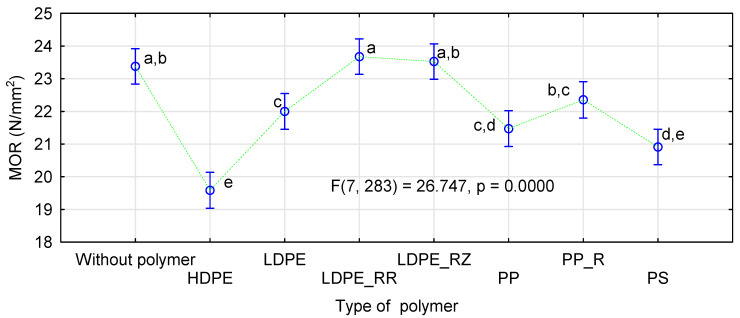
ANOVA evaluating the influence of a polymer type on static bending strength of polymer-straw boards.

The boards with PP or PS addition are characterized by relatively high strength. For these polymers, mean values of board MOR differ by 1 N/mm^2^. The boards with the lowest MOR were obtained when HDPE was used in the face layers. In this case, the strength is still high, slightly below 20 N/mm^2^, regardless of the kind of straw used. When a more extensive statistical analysis was conducted, it was found that the strength of the boards containing HDPE did not differ significantly from those containing PS (Figure 5). Furthermore, the boards manufactured with native LDPE are characterized by static bending strength statistically similar to the boards containing polypropylene. What is significant is that the boards manufactured with recycled polymers had a higher static bending strength than those containing the same polymer, but of virgin origin. Although these differences were not statistically significant in the boards with PP, the LDPE boards containing recycled polymer demonstrated improved bending strength.

**Figure 6:**
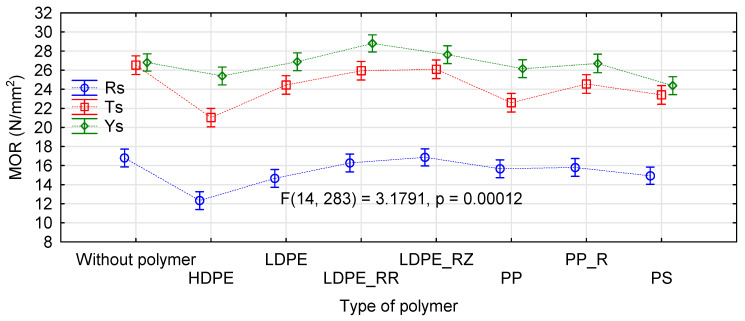
A chart presenting effects of interactions between a straw kind and a polymer type on static bending strength of polymer-straw boards.

Figure 6 presents a chart for these interactions. The interaction effect is understood as a simultaneous influence of several factors on a dependent variable in the analysis of variance. In our case, the null hypothesis about interactions must be rejected. This means that the thermoplastic polymers used in the study affected the quality of the manufactured boards always in the same way, regardless of the kind of straw used. Although the straws used in the study differed, especially the rape straw from the rye or triticale straw, none of the polymers changed significantly a trend in strength of the manufactured boards. In all cases, the boards with rye straw achieved the best, and the boards with rape straw the worst results.

**Figure 7:**
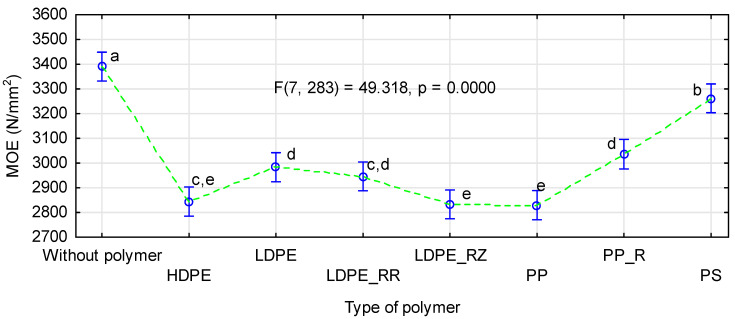
ANOVA evaluating the influence of a polymer type on modulus of elasticity of polymer-straw boards.

The effect of the straw kind on modulus of elasticity of the straw-polymer boards was similar to its influence on static bending strength (Figure 7). The best results were obtained for rye straw, and the boards with rape straw had the lowest modulus of elasticity. These differences were statistically significant (F(2, 883) = 580.55, p = 0.0000), as confirmed by the Tukey’s HSD test.

Changes in modulus of elasticity caused by the addition of a polymer to the face layers were consistent with changes in modulus of elasticity observed in previous studies [42,52,54,59]. Although, in this case, one of the lowest values of modulus of elasticity was observed also for the boards with HDPE, similarly low values were noted for the boards with LDPE or with any type of polypropylene. The lowest changes in modulus of elasticity (below 4%) were found for the boards with polystyrene. In the remaining boards, the decrease in modulus of elasticity ranged from 8% to 12%.

### 3.2. Internal Bond of Board Samples

**Figure 8:**
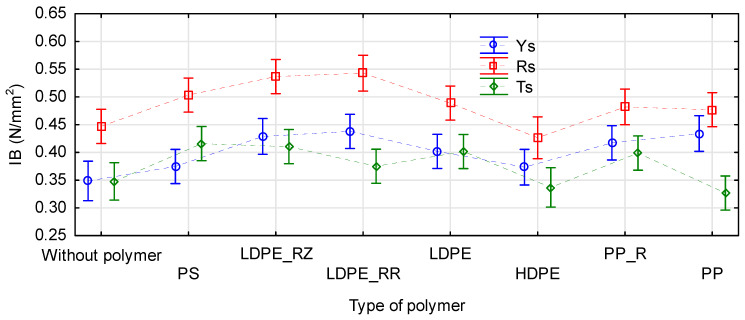
Influence of a straw kind and a polymer type on tensile strength perpendicular to the board plane.

In general, internal bond strength evaluates properties of the board core. Usually, except for special boards, in consequence of the pressing process, the density near the core of the board is the lowest, so this zone is destroyed. The boards manufactured in the study had the core of the same material, i.e., pine chips. Therefore, we could expect that the internal bond strength of all boards would be similar in this aspect. However, it turned out that there was a statistically significant difference in the manufactured boards, according to the kind of straw used in the face layers (F(2, 247) = 103.23 p = 0.0000). The tensile strength perpendicular to the board plane was the highest in the boards containing rape (mean 0.49 N/mm^2^), followed by rye (mean 0.4 N/mm^2^), and reaching the lowest value for triticale (mean 0.38 N/mm^2^). The polymer type also significant influenced the board internal bond strength (Figure 8). Internal bond strength of the boards containing HDPE was similar to that of the control boards. Slightly improved values were obtained for PP, especially for the recycled material. Internal bond strength of the boards containing polystyrene and LDPE was comparable and clearly higher than in the control boards. The best results were obtained for the recycled LDPE. In this case, the rape boards were characterized by strength exceeding 0.54 N/mm^2^.

Considering the type of gluing agent used, these boards should meet requirements for type P5 or even P7 construction boards. In terms of mechanical properties, the majority of the analyzed cases met the requirements for boards even of P7 types. Swelling of particle or straw boards during first 24 h depends, to a large extent, on added agents increasing board hydrophobic properties. Therefore, evaluation of swelling of the manufactured boards against EN 312 [70] requirements was difficult, as no improving agent was used. However, the V100 test was the most important parameter. The manufactured boards did not meet the requirements of this test. The majority of the boards achieved mean values of 0.13 N/mm^2^; however, for type P5 boards to pass this test, the 5th percentile value of 0.14 N/mm^2^ is required. As it was expected, no influence of the polymer type (F(7, 264) = 1.1786, p = 0.31524) on tensile strength perpendicular to the plane after the boiling test was observed.

### 3.3. Thickness Swelling and Water Absorption of Board Samples

**Figure 9:**
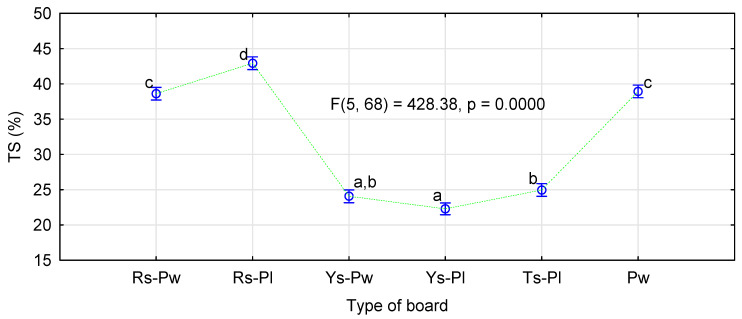
The effect of board production method and type of material on board swelling following 24 h immersion in water.

Another significant factor of the board quality is their thickness swelling after immersion in water. In this case, the applied modification did not affect observed relations (Figure 9). In general, the boards made of cereal straw showed significantly lower swelling than those made of pine chips. In this case, swelling of the straw boards, both 3- and 5-layer versions, was ca. 15% lower than of the boards made of pine chips.

The boards made of rape particles were characterized by swelling similar or slightly higher than those made of pine chips. Usually, boards made of rape particles show slightly lower swelling than pine boards; therefore, the fact that, in this study, the boards of rape particles, especially the 5-layer boards, achieved slightly higher swelling than pine boards was rather unexpected. This difference amounted to 4% against the rape boards.

**Figure 10:**
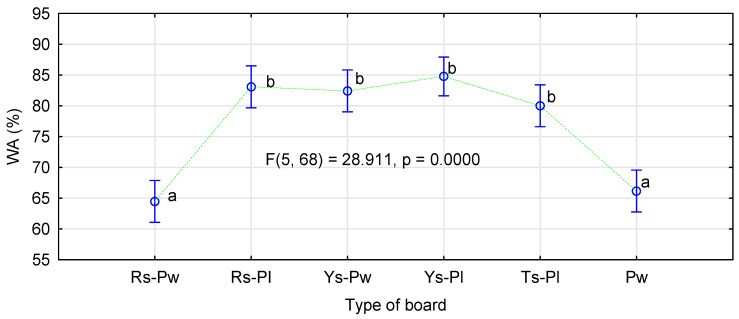
The effect of board production method and type of material on absorption capacity following 24 h immersion in water.

To some extent, high swelling of rape boards with a modified moisture content system can be explained by their high absorption capacity (Figure 10). They presented nearly 20% higher absorption capacity and, therefore, by 4% greater swelling, did not seem so important.

**Figure 11:**
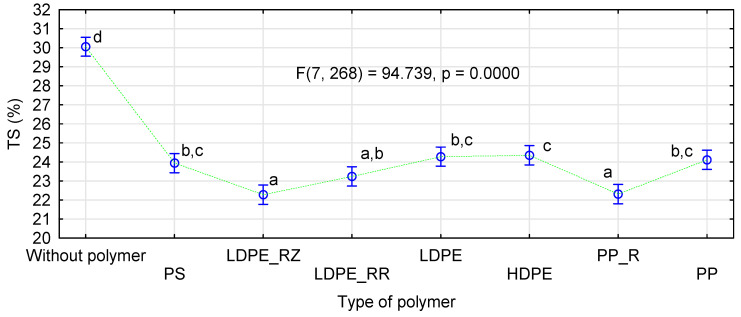
ANOVA evaluating the influence of polymer type on swelling of polymer-straw boards.

As in the case of modulus of elasticity, a chart presenting the effect of individual polymers was selected from the conducted ANOVA analyzing swelling of the straw-polymer boards (Figure 11). Additionally, in this case, the boards containing rye had the best results, followed by triticale, and with the worse results for the boards with the rape straw. The boards containing polymers were characterized by a much lower swelling when compared to the boards manufactured without them. Although differences in swelling between individual board types were not large, the statistical analysis showed that the use of recycled polymers was the most advantageous. The lowest and similar swelling values were obtained for the boards with LDPE-RZ, PP_R, and LDPE_RR. Of the boards containing polymers, those containing HDPE achieved the worst results.

**Figure 12:**
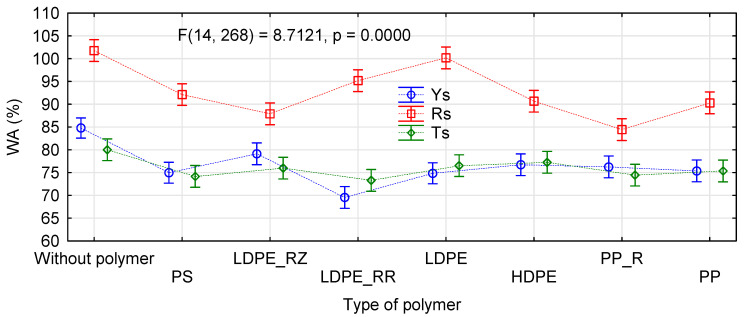
ANOVA evaluating the influence of polymer type on absorption capacity of polymer-straw boards.

The absorption capacity is usually not specified for furniture and construction boards. However, as these solutions are relatively new, Figure 12 presents a chart of interactions obtained by ANOVA. The result of the analysis itself is less important, as it is consistent with previous analyses concerning mechanical properties. However, the chart shows that the kind of straw had a lower influence on absorption capacity of the manufactured boards. As in previous studies, the boards made of rape straw absorbed much more water than the remaining straws. However, no clear difference in absorption capacity was observed between the boards made of rye and triticale straws.

## 4. Conclusions

The addition of thermoplastic polymers to board face layers significantly influenced physical and mechanical properties of manufactured boards. On one hand, improved water resistance could be observed, manifested as lower swelling or reduced water absorption capacity, while, on the other, a decrease in bending strength or modulus of elasticity was seen. However, changes in physical and mechanical properties strongly depended both on the kind of straw and the type of polymer used. The most advantageous changes in MOE and MOR were observed for the variant containing rye straw, while better results for tensile strength perpendicular to the board plane was noted with rye straw. 

The most advantageous results of modulus of elasticity and bending strength determinations were obtained for the boards containing polystyrene and recycled polymers. Changes in water resistance were achieved when recycled polyethylene or polypropylene were used. Therefore, it can be assumed that recycled polymers more strongly improve hydrophobic properties of the boards, with a smaller deteriorating effect on their mechanical properties. The structure of the straws used, although significantly different from the wood, still allows to produce the boards having good mechanical and physical properties, when they are combined with polymers. Furthermore, cereal straws allowed for better shaping of the structure, so the board bending strength was statistically higher than in boards of wood particles. Therefore, changes more frequently observed in boards containing polymers (significant drop in MOR) were not as high barrier in the case of the straw-polymer boards. 

## Figures and Tables

**Table 1:** Linear dimensions and bulk density of straw and wood particles used in the study.

Material	Symbol	Properties of Particles			
		Length (mm)	Width (mm)	Thickness (mm)	Bulk Density (kg/m^3^)
Rape straw (*Brassica napus* L. var. napus)	Rs	14.06 ± 9.8	2.18 ± 1.08	0.62 ± 0.43	80 ± 2.0
Triticale straw (*Triticosecale Witt* b m.)	Ts	18.26 ± 12.5	2.55 ± 1.25	0.30 ± 0.25	60 ± 1.2
Rye straw (*Secale* L.)	Ys	16.81 ± 11.6	1.70 ± 0.79	0.25 ± 0.17	60 ± 2.3
Pine wood particles (*Pinus sylvestris* L.)	Pw	13.72 ± 11.1	2.03 ± 1.10	1.04 ± 0.52	130 ± 1.9

**Table 2:** Basic properties of the investigated thermoplastics.

ThermoPlAstic Type	Density [g/cm^3^]	Softening Point [Vicata (A50)]	MFR (Melt Flow Index)	Photo
PP	0.9	150 °C	4 g/10 min (230 °C/2.16 kg)	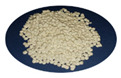
HDPE	0.952	125 °C	0.3 g/10 min (190 °C/2.16 kg)	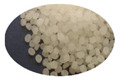
PS	1.5	90 °C	6 g/min (200 °C/5 kg)	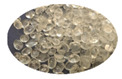
LDPE	0.923	91 °C	1.5 g/10 min (190 °C/2.16 kg)	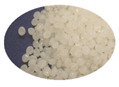
PP_R	0.54*	148 °C	Nd	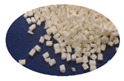
LDPE_RZ	0.37*	89 °C	Nd	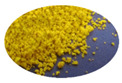
LDPE_RR	0.38 *	88 °C	Nd	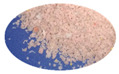

Note: *—bulk density; nd—not determined.

**Table 3:** List of boards produced and symbols used.

Board Code	Number of Board Layers	Polymer Type	Kind of Straw	Materials/Polymer Share (as %) in Board Layers				
				Layer				
				1	2	3	4	5
Pw	1	Without polymer	Without straw	Pw/0; one-layer board				
Rs-Pw	3	Without polymer	Rs	Rs/0	-	Rs/0	-	Rs/0
Ys-Pw	3	Without polymer	Ys	Ys/0	-	Ys/0	-	Ys/0
Rs-Pw-0	5	Without polymer	Rs	Rs/0	Rs/0	Pw/0	Rs/0	Rs/0
Ys-Pw-0	5	Without polymer	Ys	Ys/0	Ys/0	Pw/0	Ys/0	Ys/0
Ts-Pw-0	5	Without polymer	Ts	Ts/0	Ts/0	Pw/0	Ts/0	Ts/0
Rs-Pw-PP	5	PP	Rs	Rs/30	R/0s	Pw/0	Rs/0	Rs/30
Ys-Pw-PP	5	PP	Ys	Ys/30	Y/0s	Pw/0	Ys/0	Ys/30
Ts-Pw-PP	5	PP	Ts	Ts/30	Ts/0	Pw/0	Ts/0	Ts/30
Rs-Pw-PP_R	5	PP_R	Rs	Rs/30	R/0s	Pw/0	Rs/0	Rs/30
Ys-Pw-PP_R	5	PP_R	Ys	Ys/30	Y/0s	Pw/0	Ys/0	Ys/30
Ts-Pw-PP_R	5	PP_R	Ts	Ts/30	Ts/0	Pw/0	Ts/0	Ts/30
Rs-Pw-HDPE	5	HDPE	Rs	Rs/30	R/0s	Pw/0	Rs/0	Rs/30
Ys-Pw-HDPE	5	HDPE	Ys	Ys/30	Y/0s	Pw/0	Ys/0	Ys/30
Ts-Pw-HDPE	5	HDPE	Ts	Ts/30	Ts/0	Pw/0	Ts/0	Ts/30
Rs-Pw-LDPE	5	LDPE	Rs	Rs/30	R/0s	Pw/0	Rs/0	Rs/30
Ys-Pw-LDPE	5	LDPE	Ys	Ys/30	Y/0s	Pw/0	Ys/0	Ys/30
Ts-Pw-LDPE	5	LDPE	Ts	Ts/30	Ts/0	Pw/0	Ts/0	Ts/30
Rs-Pw-LDPE	5	LDPE	Rs	Rs/30	R/0s	Pw/0	Rs/0	Rs/30
Ys-Pw-LDPE	5	LDPE	Ys	Ys/30	Y/0s	Pw/0	Ys/0	Ys/30
Ts-Pw-LDPE	5	LDPE	Ts	Ts/30	Ts/0	Pw/0	Ts/0	Ts/30
Rs-Pw-LDPE_RZ	5	LDPE_RZ	Rs	Rs/30	R/0s	Pw/0	Rs/0	Rs/30
Ys-Pw-LDPE_RZ	5	LDPE_RZ	Ys	Ys/30	Y/0s	Pw/0	Ys/0	Ys/30
Ts-Pw-LDPE_RZ	5	LDPE_RZ	Ts	Ts/30	Ts/0	Pw/0	Ts/0	Ts/30
Rs-Pw-LDPE_RR	5	LDPE_RR	Rs	Rs/30	R/0s	Pw/0	Rs/0	Rs/30
Ys-Pw-LDPE_RR	5	LDPE_RR	Ys	Ys/30	Y/0s	Pw/0	Ys/0	Ys/30
Ts-Pw-LDPE_RR	5	LDPE_RR	Ts	Ts/30	Ts/0	Pw/0	Ts/0	Ts/30
Rs-Pw-PS	5	PS	Rs	Rs/30	R/0s	Pw/0	Rs/0	Rs/30
Ys-Pw-PS	5	PS	Ys	Ys/30	Y/0s	Pw/0	Ys/0	Ys/30
Ts-Pw-PS	5	PS	Ts	Ts/30	Ts/0	Pw/0	Ts/0	Ts/30

## Data Availability

The data presented in this study are available on request from the corresponding author.

## References

[1] Geimer R.L., Montrey H.M., Lehmann W.F. (1975). Effects of Layer Characteristics on the Properties of Three-Layer Particleboards. For. Prod. J..

[2] Moriarty C.J. (2002). The Effect of Lab-Made Flakes on Physical and Mechanical Property Variability of Laboratory Flakeboard. For. Prod. J..

[3] Mayers K.L. (2001). Impact of Strand Geometry and Orientation on Mechanical Properties of Strand Composites. Master’s Thesis.

[4] Barnes D. (2000). An Integrated Model of the Effect of Processing Parameters on the Strength Properties of Oriented Strand Wood Products. For. Prod. J..

[5] Barnes D. (2001). A Model of the Effect of Strand Lenght and Strand Thickness on the Strenght Properties of Oriented Wood Composites. For. Prod. J..

[6] Chen S., Du C., Wellwood R. (2008). Analysis of Strand Characteristics and Alignment of Commercial OSB Panels. For. Prod. J..

[7] Mirski R., Dukarska D., Derkowski A., Czarnecki R., Dziurka D. (2020). By-Products of Sawmill Industry as Raw Materials for Manufacture of Chip-Sawdust Boards. J. Build. Eng..

[8] Mirski R., Derkowski A., Dziurka D., Dukarska D., Czarnecki R. (2019). Effects of a Chipboard Structure on Its Physical and Mechanical Properties. Materials.

[9] Mirski R., Derkowski A., Dziurka D. (2018). Possibility of Using Fine Wood Strands for the Production of p5 Type Building Boards. BioResources.

[10] Moya L., Tze W.T.Y., Winandy J.E. (2009). The Effect of Cyclic Relative Humidity Changes on Moisture Content and Thickness Swelling Behavior of Oriented Strandboard. Wood Fiber Sci..

[11] Fakhri H.R., Semple K.E., Smith G.D. (2006). Transverse Permeability of OSB.; Part I. The Effects of Core Fines Content and Mat Density on Transverse Permeability. Wood Fiber Sci..

[12] Fakhri H.R., Semple K.E., Smith G.D. (2006). Transverse Permeability of OSB. Part II. Modeling the Effects of Density and Core Fines Content. Wood Fiber Sci..

[13] Han G., Wu Q., Lu J.Z. (2007). The Influence of Fines Content and Panel Density on Properties of Mixed Hardwood Oriented Strandboard. Wood Fiber Sci..

[14] Han G., Wu Q., Lu J.Z. (2006). Selected Properties of Wood Strand and Oriented Strandboard from Small Diameter Southern Pine Trees. Wood Fiber Sci..

[15] Lee L., Tahir P.M. Effects of Fine Particle Content on the Properties of Five-Layered Oriented Strand Board. Proceedings of the XII World Forestry Congress.

[16] Mirski R., Dziurka D. (2011). Applicability of Strand Substitution in the Core of OSB. BioResources.

[17] Mirski R., Dziurka D. (2011). The Utilization of Chips from Comminuted Wood Waste as a Substitute for Flakes in the Oriented Strand Board Core. For. Prod. J..

[18] Mirski R., Dziurka D., Derkowski A. (2012). The Effect of Mass Fraction of Chips Designed for Particle Board Production in the Core on Properties Of OSB. Lignocellulose.

[19] Zhang Y., Lu X., Pizzi A., Delmotte L. (2003). Wheat Straw Particleboard Bonding Improvements by Enzyme Pretreatment. Holz Roh Werkst..

[20] Boquillon N., Elbez G., Schönfeld U. (2004). Properties of Wheat Straw Particleboards Bonded with Different Types of Resin. J. Wood Sci..

[21] Bekhta P., Korkut S., Hiziroglu S. (2013). Effect of Pretreatment of Raw Materials on Properties of Particleboards Panels Made from Wheat Straw. BioResources.

[22] Hafezi S.M., Hosseini K.D. (2014). Surface Characteristics and Physical Properties of Wheat Straw Particleboards with UF Resin. J. Indian Acad. Wood Sci..

[23] Dziurka D., Mirski R. (2013). Lightweight Boards from Wood and Rape Straw Particles. Drewno.

[24] Huang L., Xia P., Liu Y., Fu Y., Jiang Y., Liu S., Wang X. (2016). Production of Biodegradable Boards Using Rape Straw and Analysis of Mechanical Properties. BioResources.

[25] Li X., Cai Z., Winandy J.E., Basta A.H. (2010). Selected Properties of Particleboard Panels Manufactured from Rice Straws of Different Geometries. Bioresour. Technol..

[26] Zhang L., Hu Y.C. (2014). Novel Lignocellulosic Hybrid Particleboard Composites Made from Rice Straws and Coir Fibers. Mater. Des..

[27] Kurochi Y., Sato M. (2015). Effect of Surface Structure, Wax Silica on the Properties of Binderless Board Made from Rice Straw. Ind. Crop. Prod..

[28] Wu T., Wang X., Kito K. (2015). Effects of Pressures on the Mechanical Properties of Corn Straw Bio-Boards. Eng. Agric. Environ. Food.

[29] Mirski R., Dziurka D., Banaszak A. (2018). Properties of Particleboards Produced from Various Lignocellulosic Particles. BioResources.

[30] Dukarska D., Czarnecki R., Dziurka D., Mirski R. (2017). Construction Particleboards Made from Rapeseed Straw Glued with Hybrid pMDI/PF Resin. Eur. J. Wood Prod..

[31] Wasylciw W. (2001). Oriented Split Straw Board—A New Era in Building Products.

[32] Cheng W., Han G., Fang D. (2013). Oriented Structural Boards from Split Wheat Straw: Effects of Straw Length, Panel Density, and Resin Content. BioResources.

[33] Mirski R., Dziurka D., Czarnecki R. (2016). The Possibility of Replacing Strands in the Core Layer of Oriented Strand Board by Particles from the Stems of Rape (*Brassica napus* L. var. napus). BioResources.

[34] Pinto J., Paiva A., Varum H., Costa A., Cruz D., Pereira S., Fernandes L., Tavares P., Agarwal J. (2011). Corn’s Cob as a Potential Ecological Thermal Insulation Material. Energ. Build..

[35] Faustino J., Pereira L., Soares S., Cruz D., Paiva P., Varum H., Ferreira J., Pinto J. (2012). Impact Sound Insulation Technique Using Corn Cob Particleboard. Constr. Build. Mater..

[36] Pinto J., Vieira B., Pereira H., Jacinto C., Vilela P., Paiva A., Pereira S., Cunha V., Varum H. (2012). Corn Cob Lightweight Concrete for Non-Structural Applications. Constr. Build. Mater..

[37] Grigoriou A.H. (2000). Straw-Wood Composites Bonded with Various Adhesive Systems. Wood Sci. Technol..

[38] Mo X.-Q., Cheng E., Wang D., Sun S. (2003). Physical Properties of Medium-Density Wheat Straw Particleboard Using Different Adhesives. Ind. Crop. Prod..

[39] Dziurka D., Mirski R. (2010). UF-pMDI Hybrid Resin for Waterproof Particleboards Manufactured at a Shortened Pressing Time. Drvna Ind..

[40] Mirski R., Boruszewski P., Trociński A., Dziurka D. (2018). The Possibility to Use Long Fibres from Fast Growing Hemp (*Cannabis sativa* L.) for the Production of Boards for the Building and Furniture Industry. BioResources.

[41] http://vestaeco.pl/index.html.

[42] Borysiak S., Paukszta D. (2008). Mechanical Properties of Lignocellulosic/Polypropylene Composites. Mol. Cryst. Liq. Cryst..

[43] Markiewicz E., Paukszta D., Borysiak S. (2019). Dielectric Properties of Lignocellulosic Materials-Polypropylene Composites. Mater. Sci. Poland.

[44] Niska K., Sain M. (2008). Wood-Polymer Composites.

[45] Wolcott M.P. (2003). Formulation and Process Development of Flat Pressed Wood-Polyethylene Composites. For. Prod. J..

[46] Ayrilmis N., Jarusombuti S. (2011). Flat-Pressed Wood Plastic Composite as an Alternative to Conventional Wood-Based Panels. J. Compos. Mater..

[47] Benthien J.T., Thoemen H. (2012). Effects of Raw Materials and Process Parameters on the Physical and Mechanical Properties of Flat Pressed WPC Panels. Compos. Part A Appl. Sci. Manuf..

[48] Schmidt H., Benthien J., Thoemen H. (2013). Processing and Flexural Properties of Surface Reinforced Flat Pressed WPC Panels. Eur. J. Wood Prod..

[49] Lyutyy P., Bekhta P., Sedliačik J., Ortynska G. (2014). Properties of Flat-Pressed Wood-Polymer Composites Made Using Secondary Polyethylene. Acta Fac. Xylologiae Zvolen.

[50] Bekhta P., Lyutyy P., Ortynska G. (2016). Effects of Different Kinds of Coating Materials on Flat Pressed WPC Panels. Drvna Ind..

[51] Bekhta P., Lyutyy P., Ortynska G. (2017). Properties of Veneered Flat Pressed Wood Plastic Composites by One-Step Process Pressing. J. Polym. Environ..

[52] Mirski R., Dziurka D., Banaszak A. (2019). Using Rape Particles in the Production of Polymer and Lignocellulose Boards. BioResources.

[53] Synowiec J. (1996). Sposób Wytwarzania Kompozytu z Polimerów Termoplastycznych i Cząstek Lignocelulozowych.

[54] Borysiuk P., Mamiński M., Zado A. (2009). Some Comments on the Manufacturing of Thermoplastic-Bonded Particleboards. Ann. Warsaw Univ. Life Sci. For. Wood Technol..

[55] Hague J.R.B., Loxton C., Quinney R., Hobson N. Assessment of the Suitability of Agri-Based Materials for Panel Products. Proceedings of the First European Panel Products Symposium.

[56] Papadopoulos A.N., Hague J.B.B. (2003). The Potential for Using Flax (*Linum usitatissimum* L.) Shiv as a Lignocellulosic Raw Material for Particleboard. Ind. Crop. Prod..

[57] Directive 2008/98/Ec of the European Parliament and of the Council of 19 November 2008 on Waste and Repealing Certain Directives. https://eur-lex.europa.eu.

[58] Chaharmahali M., Mirbagheri J., Tajvidi M., Najafi S.K., Mirbagheri Y. (2010). Mechanical and Physical Properties of Wood-Plastic Composite Panels. J. Reinf. Plast. Comp..

[59] Mirski R., Dziurka D., Bekhta P. (2019). Relationships between Thermoplastic Type and Properties of Polymer-Triticale Boards. Polymers.

[60] Borysiuk P. (2012). MożLiwośCI Wytwarzania PłYT WIórowo-Polimerowych Z Wykorzystaniem PoużYtkowych Termoplastycznych Tworzyw Sztucznych.

[61] Qi C., Yadama V., Guo K., Wolcott M. (2013). Thermal Conductivity of Sorghum and Sorghum–Thermoplastic Composite Panels. Ind. Crop. Prod..

[62] Research Report (2003). Wood Plastic Composites Study-Technologies and UK Market Opportunities.

[63] Dziurka D., Mirski R., Łęcka J. (2006). The Effect of Pine Particle Moisture Content on Properties of Particleboards Resinated with PMDI. EJPAU.

[64] Park B.D., Riedl B., Hsu E.W., Shields J. (1999). Hot-Pressing Process Optimization by Response Surface Methodology. For. Prod. J..

[65] Kelly M.W. (1977). Critical Literature Review of Relationships between Processing Parameters and Physical Properties of Particleboard. General Technical Report FPL-10.

[66] EN 310 (1993). Wood-Based Panels—Determination of Modulus of Elasticity in Bending and of Bending Strength.

[67] EN 319 (1993). Particleboards and Fibreboards—Determination of Tensile Strength Perpendicular to the Plane of the Board.

[68] EN-1087-1 (1995). Particleboards—Determination of Moisture Resistance—Boil Test.

[69] EN 317 (1993). Particleboards and Fibreboards. Determination of Swelling in Thickness after Immersion in Water.

[70] EN 312 (2003). Particleboards—Specifications.

